# Drug resistance patterns following pharmacy stock shortage in Nigerian Antiretroviral Treatment Program

**DOI:** 10.1186/s12981-017-0184-5

**Published:** 2017-10-13

**Authors:** Seema T. Meloni, Beth Chaplin, John Idoko, Oche Agbaji, Sulaimon Akanmu, Godwin Imade, Prosper Okonkwo, Robert L. Murphy, Phyllis J. Kanki

**Affiliations:** 1 Department of Immunology & Infectious Diseases, Harvard T. H. Chan School of Public Health, 651 Huntington Avenue, FXB 405, Boston, 02115 MA USA; 2 University of Jos, Jos, Plateau State Nigeria; 3 Jos University Teaching Hospital, Jos, Plateau State Nigeria; 4 Lagos University Teaching Hospital, Lagos, Lagos State Nigeria; 5 APIN Public Health Initiatives, Ltd./Gte, Abuja, Nigeria; 6 Northwestern University, Chicago, IL USA

**Keywords:** Antiretroviral therapy, Drug resistance, Mutations, Unstructured treatment interruption, Drug shortage, Nigeria

## Abstract

**Background:**

For patients on antiretroviral therapy (ART), treatment interruptions can impact patient outcomes and result in the accumulation of drug resistance mutations leading to virologic failure. There are minimal published data on the impact of an ART stock shortage on development of drug resistance mutations (DRMs). In this report, we evaluate data from patients enrolled in the Government of Nigeria National ART Program that were receiving treatment at the time of a national drug shortage in late 2003.

**Methods:**

We conducted a cross-sectional evaluation of samples collected between December 2004 and August 2005 from ART patients in virologic failure that either had a treatment interruption or did not during the late 2003 drug shortage period at the Jos University Teaching Hospital (JUTH). Plasma virus was genotyped, sequence data were edited and analyzed, and mutation profiles were categorized to evaluate predicted drug susceptibility. Data were analyzed to examine factors associated with development of resistance mutations. A genotypic sensitivity score to the alternate recommended regimen was computed to assess drug susceptibility if regimens were changed.

**Results:**

A total of 56 patients were included in this evaluation (28 interrupted, 28 uninterrupted). Patients in the interrupted group had more DRMs than those in the uninterrupted group (p < 0.001); interrupted patients were more likely than uninterrupted patients to have one or more TAM-2 mutations (57.1% interrupted vs. 21.3% uninterrupted; p = 0.04). There was a statistically significant difference in resistance to both d4T (53.7% interrupted vs. 17.9 uninterrupted; p = 0.011) and AZT (64.3% interrupted vs. 25.0% uninterrupted; p = 0.003) by drug interruption status. Examining genotypic sensitivity scores, we found that 67.9% of the interrupted patients, as compared to 25.0% of the uninterrupted patients, did not have full susceptibility to one drug in the regimen to which guidelines recommended they be switched (p = 0.001).

**Discussion:**

In this small observational study, we found evidence of a difference in resistance profiles and ART susceptibility between those that were stocked-out of drug versus those that were not. We believe that these data are relevant for many other low- and middle-income countries (LMIC) that also experienced similar ART shortages as they rapidly scaled up their national programs.

## Introduction

It is well documented that treatment interruptions (TI), both structured and unstructured (uTI), have an impact on antiretroviral therapy (ART) outcomes and can result in the accumulation of drug resistance mutations (DRMs) leading to virologic failure (VF) [1]. The majority of TI studies focus on cohorts where either a clinician or patient was responsible for the TI and largely examine predictors of the TI. To our knowledge, there are no published data on the impact of a drug shortage on development of DRMs, where mutation data from patients who did not receive the ART due to the shortage (i.e., interrupted) are compared to those of patients on treatment at the same time that were not affected by the shortage (i.e., uninterrupted). In this report, we present data from patients enrolled in the Government of Nigeria (GoN) National ART Program that were receiving treatment at the time of a national drug shortage. The shortage occurred during the early period of the establishment of Nigeria’s National ART Program, a situation that likely occurred in many other low- and middle-income countries (LMIC) as they were scaling up their national programs.

Starting in early 2002, the GoN initiated a National ART Program to treat 10,000 adults and 5000 children infected with human immunodeficiency virus (HIV). The GoN established 26 treatment centers across the country and patients were initiated on a combination of stavudine (d4T) + lamivudine (3TC) + nevirapine (NVP) at the subsidized rate of 1000 Naira (USD $8, at the time). While there was a rapid scale-up of initiating patients on ART, between November 2003 and January 2004, the program experienced an unexpected ART stock shortage, which affected an estimated 8000 patients. Treatment was resumed once the drug supplies were replaced, but the impact of that uTI had not been evaluated. In this study, we analyzed DRMs among HIV-infected individuals that eventually failed ART, stratifying between those that experienced an interruption of ART during the drug shortage period and those that continued to receive their ART.

## Methods

### Study cohort

We conducted a cross-sectional evaluation of samples from ART patients that experienced VF between December 2004 and August 2005 at the Jos University Teaching Hospital (JUTH), a Harvard/AIDS Prevention Initiative Nigeria (APIN) Presidents Emergency Plan for AIDS Relief (PEPFAR) Program-supported GoN ART site; all patients had enrolled for ART between June 2004 and June 2005. Interrupted patients were those that did not receive their regular medications between November 2003 and January 2004, while uninterrupted patients were those that were able to receive their regular medication during the same time period. Inclusion criteria for this evaluation consisted of: enrollment in National Program prior to January 2004, ≥ 12 months on ART, age ≥ 18 years, and viral load (VL) count ≥ 10,000 copies (cp)/mL at or after VF.

### Data collection

Plasma virus from patients in VF was genotyped in the reverse transcriptase (RT) and protease (PR) genes using the ViroSeq HIV-1 Genotyping System 2.0 Assay (Abbott, Chicago, IL). All sequence data were edited and analyzed to generate lists of mutations and polymorphisms. Mutations were categorized according to the International Antiviral Society (IAS)-USA recommendations [2]. Drug susceptibility was based on the Stanford University HIV Drug Resistance Database (HIVdb) algorithm [3, 4].

Following enrollment in the Harvard/APIN PEPFAR-supported GoN program, patient data were collected and stored using an electronic medical records system (EMRS) [5]. For the statistical evaluations, patient data, including ART regimen, date of birth (for age calculation), sex, occupation, education, marital status, WHO stage at entry, HIV subtype, CD4+ cell count at program entry and at sample dates, VL at entry and sample dates, and adherence were abstracted from the EMRS. Time of ART initiation was defined as the date the patient initially started taking ART in the GoN program, time of program entry was defined as the date the patient enrolled in the Harvard/APIN PEPFAR-supported ART program at JUTH, and sample date was defined as the date the patient’s sample was collected or DRM testing (following program entry). VL was stratified based commonly accepted clinical categories: < 400; 400–999; 1000–9999; 10,000–99,999; and ≥ 100,000 cp/mL (i.e., high VL).

### Statistical analysis

Patient characteristics at the time of program enrollment were compared for interrupted versus uninterrupted patients using bivariate methods, including Wilcoxon rank-sum test for continuous variables and Chi square or Fisher’s exact tests for categorical variables, as relevant. We examined the potential association between HIV-1 subtype to DRMs and resistance using the Fisher’s exact test; for statistical analyses, A1, A3, CRF06_cpx and unknown were combined to make an “other” category. To assess if adherence patterns could potentially explain the difference in VL values at sample date, we evaluated the association using Fisher’s exact test. To evaluate the predicted impact of substituting the d4T+3TC+NVP regimen to the recommended alternative regimen of zidovudine (AZT)+3TC+efavirenz (EFV), for patients failing at that time, a genotype susceptibility score (GSS) to the alternative recommended regimen was calculated. GSS was computed based on the drug resistance scores extracted from the Stanford HIVdb. Each ARV drug in the alternative recommended regimen was assigned a score according to the five-level Stanford HIVdb interpretation: 1.00 for susceptible, 0.75 for potential low-level resistance, 0.50 for low-level resistance, 0.25 for intermediate resistance and 0.0 for high-level resistance [6]. The GSS was the sum of all scores. Percentage of patients with a low GSS were compared by drug interruption status using the Wilcoxon rank-sum test. Due to the nature of the small sample size and the fact that we were underpowered to generate a multivariate model on the data, we also conducted a stratified analysis to compare GSS by drug interruption status in only those patients who had < 12 months between entry and their sample date. All statistical analyses were performed using Stata 13.1 (College Station, TX).

### Ethical considerations

All patients provided written informed consent for inclusion in the cohort. This study was approved by the Institutional Review Boards at JUTH and the Harvard T. H. Chan School of Public Health.

## Results

### Study cohort

In total, there were 56 patients included in this evaluation: 28 that experienced interruption and 28 that did not. We found that patients in the interrupted group only differed from the uninterrupted group in gender breakdown, time from ART initiation to sample date, and time from program entry to time of sample date. There was a higher percentage of males in the interrupted group as compared to the uninterrupted group (57.1% vs. 21.4%; p = 0.006; Table 1). We found that the time from both ART initiation (AI) and program entry to sample date was longer in the interrupted patients versus uninterrupted patients (AI to sample date: 365 vs. 213 days; entry to sample: 1036 vs. 601 days; p < 0.0001). At the time of sampling, 14.3% of the interrupted patients versus 10.7% of the uninterrupted patients had VL ≥ 100,000 cp/mL (p < 0.001). While 100% of the patients in the interrupted group versus 35.7% of those in the uninterrupted group had VL ≥ 10,000 at the sampling date, we found no association between VL at sample date and adherence patterns during the time period of program entry to VL sampling date; the data indicated that 72.7% of patients with an average adherence of < 95% had VL ≥ 10,000 cp/mL compared to 64.7% of patients with an average adherence of ≥ 95% (p = 0.57; data not shown). Median CD4+ cell count at sample date for the interrupted patients was 123 cells/mL as compared to 257 cells/mL for the uninterrupted patients (p = 0.004). The majority of the patients in the cohort did not achieve an undetectable VL following enrollment in the program and there was no difference by drug interruption status.

**Table 1:** Baseline demographic and clinical characteristics of included patients

	Total	Drug regimen interruption		p value
		Yes	No	
Sex, n (%)				0.006
Female	34 (60.7)	12 (42.9)	22 (78.6)	
Male	22 (39.3)	16 (57.1)	6 (21.4)	
Median age (IQR)	37 (31.5–45.0)	39.5 (34.5–45.5)	36.0 (29.5–41.5)	0.08
Education, n (%)				0.14
Primary	7 (12.5)	2 (7.1)	5 (17.9)	
Secondary	21 (37.5)	14 (50.0)	7 (25.0)	
Tertiary	28 (50.0)	12 (42.9)	16 (57.1)	
Occupation type, n (%)				0.30
Non-income generating	10 (17.9)	3 (10.7)	7 (25.0)	
Income-generating	46 (82.1)	25 (89.3)	21 (75.0)	
WHO stage at entry				0.41
1	24 (42.9)	15 (53.6)	9 (32.1)	
2	18 (32.1)	7 (25.0)	11 (39.3)	
3	12 (21.4)	5 (17.9)	7 (25.0)	
4	2 (3.6)	1 (3.6)	1 (3.6)	
Subtype				0.07
A1	3 (5.4)	2 (7.1)	1 (3.6)	
A3	1 (1.8)	0 (0.0)	1 (3.6)	
G	9 (16.1)	3 (10.7)	6 (21.4)	
G’	15 (26.8)	4 (14.3)	11 (39.3)	
CRF02_AG	23 (41.1)	16 (57.1)	7 (25.0)	
CRF06_cpx	1 (1.8)	1 (3.6)	0 (0.0)	
Unknown	4 (7.1)	2 (7.1)	2 (7.1)	
CD4+ Count at entry, n (%) (cells/mL)				0.62
< 100	14 (25.0)	8 (28.6)	6 (21.4)	
100–199	17 (30.4)	9 (32.1)	8 (28.6)	
200–349	15 (26.8)	8 (28.6)	7 (25.0)	
≥ 350	10 (17.9)	3 (10.7)	7 (25.0)	
VL at entry, n (%) (cp/mL)				0.58
< 400 (undetectable)	12 (21.4)	5 (17.9)	7 (25.0)	
400-999	6 (10.7)	3 (10.7)	3 (10.7)	
1000-9999	8 (14.3)	5 (17.9)	3 (10.7)	
10,000-99,999	18 (32.1)	11 (39.3)	7 (25.0)	
≥ 100,000	12 (21.4)	4 (14.3)	8 (28.6)	
Days from program entry to sample, median (IQR)	362 (196–365)	365 (364–378)	213 (168–340)	< 0.0001
Days from ART initiation to sample, median (IQR)	745 (581–1036)	1036 (986–1107)	601 (176–704)	< 0.0001
Undetectable VL between program entry and sample dates, n (%)	15 (26.8)	6 (21.4)	9 (32.1)	0.37
VL at sample date, n (%) (cp/mL)				< 0.001
1000–9999	18 (32.1)	0 (0.0)	18 (64.3)	
10,000–99,999	31 (55.4)	24 (85.7)	7 (25.0)	
≥ 100,000	7 (12.5)	4 (14.3)	3 (10.7)	
CD4 count at sample date, median (IQR)	177 (83–301)	123 (41–216)	257 (151–360)	0.004
Average % adherence entry to sample date*, n (%)				0.11
< 70	3 (5.4)	0 (0.0)	3 (10.7)	
70–79	3 (5.4)	2 (7.1)	1 (3.6)	
80–89	8 (14.3)	5 (17.9)	3 (10.7)	
90–94	8 (14.3)	6 (21.4)	2 (7.1)	
95–99	13 (23.2)	8 (28.6)	5 (17.9)	
100	21 (37.5)	7 (25.0)	14 (50.0)	

### HIV drug resistance

**Fig. 1:**
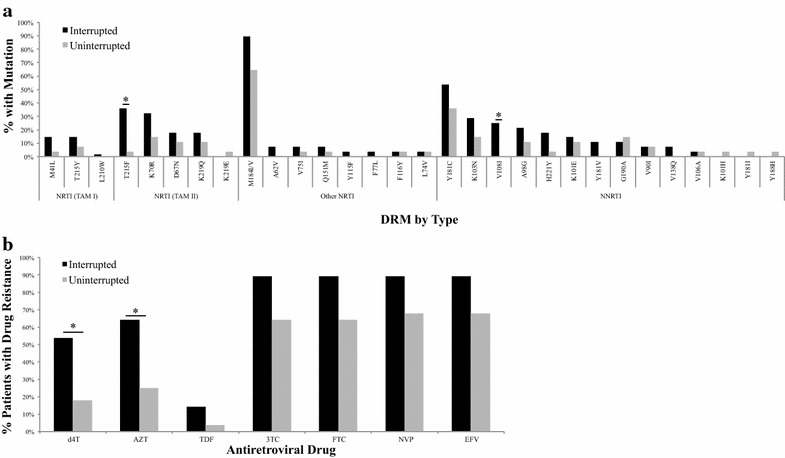
Drug resistance by stock-out status. **a** DRMs by stock-out status; **b** Drug resistance to recommended 1L antiretroviral drugs by stock-out status. * Statistically significant difference by stock-out status detected

In the interrupted arm, patients had a median of five DRMs [interquartile ratio (IQR): 3–6] compared to 3 (IQR: 0–4) in the uninterrupted arm (p = 0.001). The most common nucleoside reverse transcriptase inhibitor (NRTI) mutation for the cohort was M184I/V (76.8%), where 25 (89.3%) of the interrupted patients and 18 (64.3%) of the uninterrupted patients harbored the mutation at the time of sampling (p = 0.06; Fig. 1a). Thymidine analog mutations (TAMs) were only found in patients that harbored M184I/V (62.8% of those with M184I/V and 0% of those with no M184I/V; p < 0.001; data not shown). The only NRTI mutation for which we detected a statistically significant difference by drug interruption status was T215F, a TAM-2 (35.7% for interrupted vs. 3.6% for uninterrupted; p = 0.005; Fig. 1a). We also found that interrupted patients were more likely than uninterrupted patients to have one or more TAM-2 mutations (57.1% interrupted vs. 21.3% uninterrupted; p = 0.04). The most common non-NRTI (NNRTI) mutation was Y181C, but the difference between patient groups was not statistically significant. The only NNRTI for which we found a difference in proportion harboring the DRM by drug interruption status was V108I (25.0% interrupted vs. 0% uninterrupted; p = 0.01). The only mutation for which we detected a statistically significant difference by subtype was A98G, where patients infected with subtype G or CRF02_AG were more likely than those with G-prime or any other mutation to have the A98G mutation (CRF02_AG: 26.1%; G: 33.3%; G-prime: 0.0%; Other (all others combined): 0.0%; p = 0.03; data not shown); however, the association did not remain once we stratified by drug interruption status (data not shown).

While the study was not powered to detect differences by drug interruption status for the majority of the individual mutations, we found that a higher percentage of patients overall were resistant to the NVP and 3TC components of their ART regimen as compared to their d4T component (Fig. 1b). The majority of patients retained susceptibility to tenofovir (TDF). We were not able to show a statistically significant difference in proportion of patients with resistance to 3TC, FTC, NVP or EFV by drug interruption status. However, we did find a statistically significant difference by drug interruption status for resistance to both d4T (53.7% interrupted vs. 17.9 uninterrupted; p = 0.011) and AZT (64.3% interrupted vs. 25.0% uninterrupted; p = 0.003). We were also not able to detect a statistically significant difference in drug resistance by HIV-1 subtype (data not shown).

The overall median GSS to alternate regimen for the study population was 1.00 (IQR: 0.25, 3.00), with the median being 1.25 (IQR: 0.75–3.00) for the interrupted group and 0.50 (IQR: 0.25–1.00) for the uninterrupted group. Using a cut-off of GSS < 1, we found that 19 (67.9%) of the interrupted patients, as compared to 7 (25.0%) of the uninterrupted patients, did not have full susceptibility to one drug in the regimen to which guidelines recommended they be switched (p = 0.001). When we limited the GSS comparison to only those patients that had < 12 months between entry into the PEPFAR program and their sample date, GSS remained 1.25 (IQR: 0.75–3.00) for the interrupted group and dropped to 0.38 (IQR: 0.25–1.25) for the uninterrupted group, where 9 (56.3%) of the interrupted patients versus 7 (25.0%) of the uninterrupted patients had susceptibility to none of the recommended drugs (p = 0.04).

## Discussion

In this evaluation, we examined impact of uTI on DRMs and drug resistance due to a three-month ART stock shortage by comparing a group of patients that had a temporary interruption of medication to a group that was being treated at approximately the same time, that were not impacted by the drug shortage. In this small, cross-sectional, observational study, we found evidence of a difference in resistance profiles between those that were stocked-out of drug versus those that were not. Technically, since all of the patients included in this evaluation experienced VF, patients in the uninterrupted arm likely also had some level of TI; however, the combined duration of TIs in the uninterrupted arm is not expected to have been as great as that of the patients in the interrupted arm. Furthermore, we found no statistically significant difference in adherence patterns between the groups during the time period between PEPFAR Program entry and their VL sampling date.

Interestingly, we observed that over three-quarters of the study arm that did not experience the treatment interruption was female. At the time of the shortage, there was no concerted effort to ensure that more women versus men remained on treatment. Therefore, we suspect that these numbers are partially a result of sampling bias and might also be explained, to some degree, by the better health-seeking behaviors of women compared to men.

Overall, the most common mutations seen in this cohort are similar to those found in other studies examining DRMs in patients failing first-line (1L) ART [7–10] and we found some differences seen in mutation profiles in the patients that were impacted by the drug shortage as compared to those that continued to receive their medications through that time period. The finding of higher percentage of patients with resistance to 3TC compared to d4T is not surprising considering resistance to 3TC is known to require a single mutation (M184I/V). The higher rate of mutation towards NVP/EFV can be explained by the data indicating that NVP has a longer half-life in the blood stream; when triple drug regimen exposure is stopped, patients are effectively retained on a suboptimal regimen of NVP alone up to an estimated 2 weeks as the other two drugs are purged from the bloodstream [11, 12]. The resistance patterns are similar to those seen in a small study from Malawi, where they examined resistance patterns in patients that were also on the 1L regimen of d4T+3TC+NVP, but had experienced varying ranges of TI [7]. Our findings are consistent with the conclusions made by Pennings [13] that long treatment interruptions carry a high risk of evolution of resistance than relatively shorter interruptions, particularly since the resistance that developed was not only towards NVP, the drug with the longer half-life, where the “tail of monotherapy” might explain the accumulation of mutations, but also to d4T and 3TC.

The findings regarding GSS are also noteworthy as we found that patients that experienced the interruption were less likely to have susceptibility to the alternate recommended regimen to which they would have been switched at the time these patients were receiving treatment. Thus, programs that were switching patients that experienced TI and were in VF from the regimen of d4T+3TC+NVP to the alternate of AZT+3TC+EFV were putting them on a suboptimal regimen to which they were likely developing subsequent additional DRMs.

As a preliminary investigation of impact of uTI due to drug shortage, our analyses were limited due to the small sample size; this evaluation was not powered to detect a difference by drug interruption status for all the various mutations evaluated and also could not include multivariate methods to examine multiple predictors of resistance. Furthermore, given that this was a cross-sectional observational evaluation, we do not have access to data regarding baseline or transmitted mutations at the time of ART initiation; however, since the patients all started treatment in either 2002 or 2003 and because the country had little access to HIV treatment prior to that time, we suspect that the majority of the patients likely had little to no transmitted mutations or resistance. As we suspect this type of information will be relevant to multiple LMIC, which are in various stages of establishing and growing National ART Programs, we recommend a larger scale study powered to examine other confounders to allow for a better understanding of impact of stock shortages on patient outcomes.

## Abbreviations

TI: treatment interruption
uTI: unstructured treatment interruption
ART: antiretroviral therapy
DRM: drug resistance mutation
VF: virologic failure
GoN: Government of Nigeria
LMIC: low- and middle-income countries
HIV: human immunodeficiency virus
d4T: stavudine
3TC: lamivudine
NVP: nevirapine
USD: United States Dollars
JUTH: Jos University Teaching Hospital
APIN: AIDS prevention initiative Nigeria
PEPFAR: President’s Emergency Plan for AIDS Relief
VL: viral load
RT: reverse transcriptase
PR: protease
HIVdb: HIV drug resistance database
IAS-USA: International Antiviral Society-USA
EMRS: electronic medical records system
CP: copies
AZT: zidovudine
EFV: efavirenz
GSS: genotypic sensitivity score
AI: ART initiation
IQR: interquartile ratio
TAM: thymidine analog mutation
NRTI: nucleoside reverse transcriptase inhibitor
NNRTI: non-NRTI
TDF: tenofovir
1L: first-line ART
